# A Case of Robot‐Assisted Radical Cystectomy for a Third Kidney Transplant Recipient

**DOI:** 10.1002/iju5.70264

**Published:** 2026-09-06

**Authors:** Fumito Yamabe, Mizuki Kasahara, Kei Sakurabayashi, Yozo Mitsui, Hideyuki Kobayashi, Koichi Nakajima

**Affiliations:** ^1^ Department of Urology Toho University Faculty of Medicine Tokyo Japan; ^2^ Department of Nephrology Toho University Faculty of Medicine Tokyo Japan

**Keywords:** bladder cancer, kidney transplant recipient (KTR), minimally invasive surgery, radical cystectomy, robot‐assisted radical cystectomy (RARC)

## Abstract

**Introduction:**

Although robot‐assisted surgery is widely used, there are few reports of robot‐assisted radical cystectomy in kidney transplant recipients. We report our experience with robot‐assisted radical cystectomy in a patient who had undergone three kidney transplants.

**Case Presentation:**

The patient was a 57‐year‐old man who had undergone three kidney transplants. Robot‐assisted total cystectomy was performed for bladder CIS. Adhesions were observed in areas that had been operated on in previous surgeries, and although there were times when the surgery was difficult, it was performed safely. No major postoperative complications were observed, and the patient was discharged from the hospital on the 19th postoperative day.

**Conclusion:**

Robot‐assisted surgery is one of the surgical techniques that many urologists are familiar with, and our findings suggest that it may be safe and effective even for such complex cases.

**Keynote Message**

- We performed robot‐assisted radical cystectomy on a patient who had undergone three kidney transplants.
- It may be possible to leverage the advantages of robot‐assisted surgery even in complex cases involving a history of multiple abdominal surgeries.

**Abbreviations**
KTR: kidney transplant recipients
RARC: robot‐assisted radical cystectomy
RARP: robot‐assisted radical prostatectomy

## 1 Introduction

In recent years, robot‐assisted surgery has become the predominant approach for radical prostatectomy and radical cystectomy to treat prostate and bladder cancers. This includes cases involving patients who have undergone kidney transplantation; while there are numerous reports regarding RARP in such patients [1, 2], reports on RARC in this population are limited. Here, we report our experience with RARC performed on a patient who had undergone three kidney transplantations.

## 2 Case Presentation

The patient is a 57‐year‐old male who has undergone three kidney transplantations. His renal failure is attributed to Alport syndrome. He has received three kidney transplants: bilateral iliac fossae and the cranial portion of the right iliac fossae. The first transplanted kidney was removed by nephrectomy. He also underwent open ileocecal resection for ascending colon cancer.

Three years ago, he was diagnosed with ureteral cancer in his right kidney following an investigation into right hydronephrosis, and underwent laparoscopic right nephroureterectomy. He has also undergone multiple transurethral resections (TUR‐Bt) for bladder cancer.

A table summarizing the history of abdominal surgeries is presented separately. (Table 1).

**TABLE 1:** History of abdominal surgeries.

Age (year)	Surgery
15	First kidney transplant (living donor) day 7 graft loss →graft nephrectomy
16	Second kidney transplant (cadaver) day 10 graft loss
29	Third kidney transplant (living donor)
54	Laparoscopic Rt nephroureterectomy (Upper urinary tract cis)
56	Open ileocecal resection (colon cancer)

**FIGURE 1:**
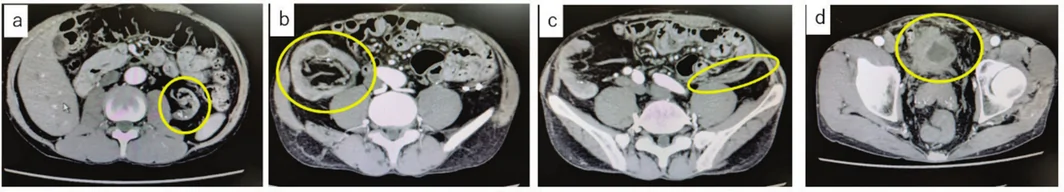
Preoperative contrast‐enhanced CT. (a) Left autologous kidney. (b) Transplanted kidney (third). (c) Transplanted kidney (second). (d) Bladder.

**FIGURE 2:**
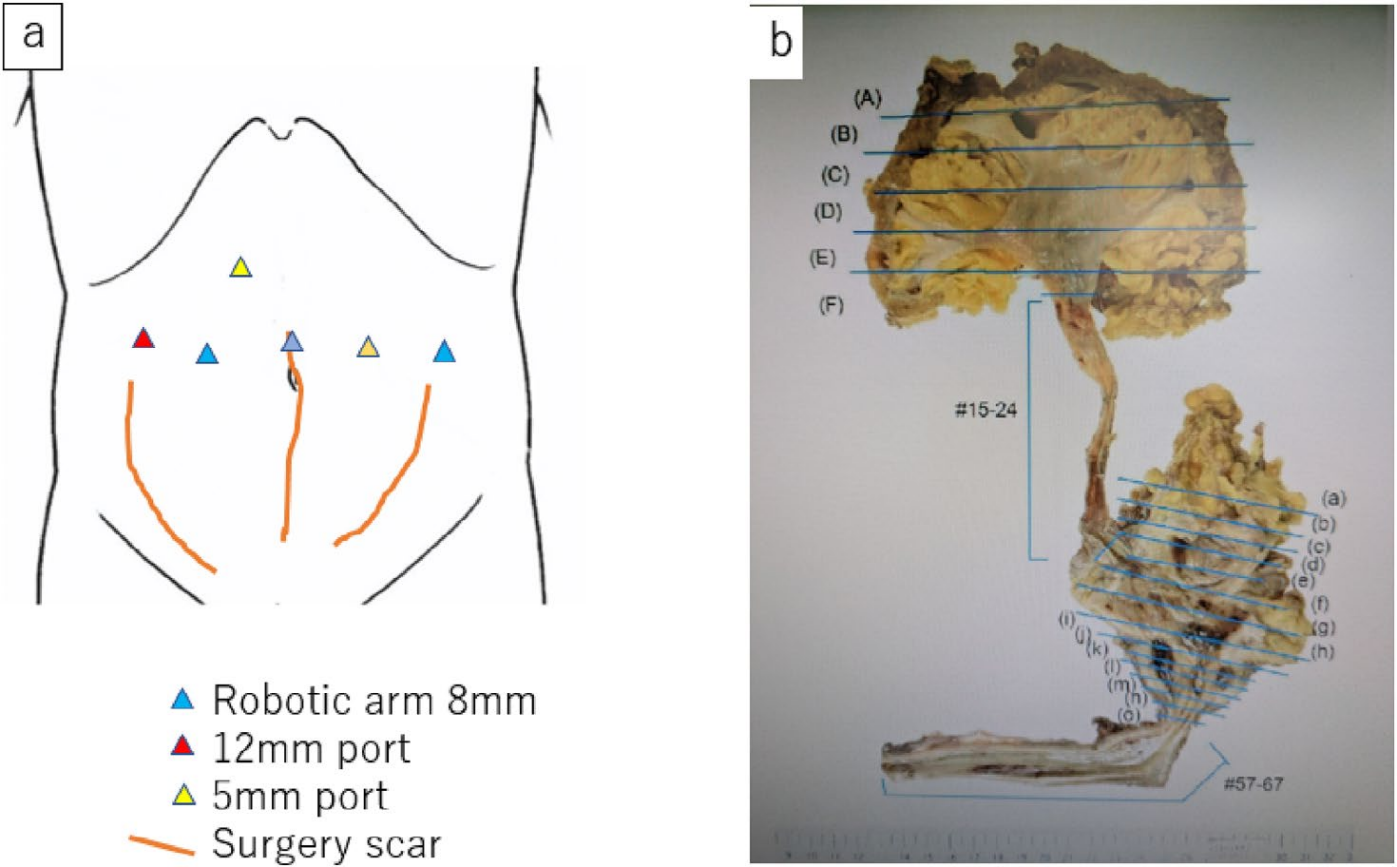
(a) Abdominal surgical scars and the port placement. (b) Excised specimen.

He was diagnosed with bladder carcinoma in situ (CIS) and underwent BCG bladder instillation therapy, but it was resistant to treatment. Therefore, he underwent RARC and open tertiary nephrectomy. Due to the deterioration of transplanted kidney function, hemodialysis was reintroduced, and urinary diversion was not performed. Preoperative contrast‐enhanced CT images are shown (Figure 1). Abdominal surgical scars and the port placement used during the procedure are shown (Figure 2a). Because the third transplanted kidney was located in the right lower abdomen, the right‐side assistant port was placed more cranially than usual.

**FIGURE 3:**
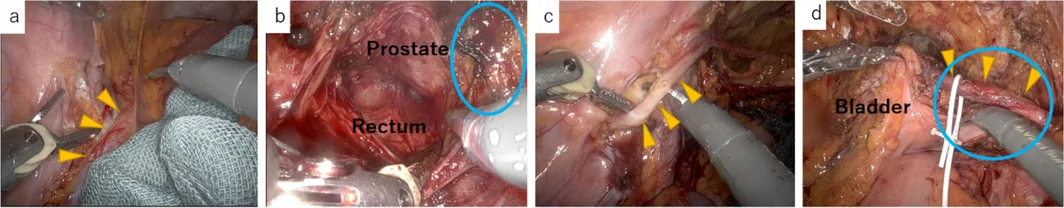
Intraoperative images. (a) Native left ureter (Yellow arrow). (b) Dissection of the posterior aspect of the prostate. Hard tissue on the right side of the bladder (Blue circle mark). Ureter of the secondary transplanted kidney (Yellow arrow). Ureter of the third transplanted kidney (Yellow arrow). Hard tissue on the right side of the bladder (Blue circle mark).

Surgical maneuvers began on the left side, where the impact of previous surgeries was expected to be relatively minimal (Figure 3). The native left ureter was identified and dissected toward the bladder (Figure 3a). After identifying the seminal vesicles bilaterally on the dorsal side of the bladder, Denonvilliers' fascia was incised to expose the dorsal aspect of the prostate (Figure 3b). We transected the native left ureter and the ureter of the secondary transplanted kidney and confirmed via intraoperative frozen section analysis that no tumor cells were present at the ureteral resection margins. Next, the Space of Retzius was developed, proceeding from the left side toward the right. During this process, the ureter of the third transplanted kidney was identified and dissected (Figure 3d). Surgical manipulation was challenging due to tissue induration around the ureter of the third transplanted kidney and the right lateral vesical vascular pedicle (Figure 3b,d). The robotic‐assisted phase of the surgery concluded with only the urethra and the ureter of the third transplanted kidney remaining attached. Urethrectomy and removal of the third transplanted kidney were performed via open surgery to extract the specimens.

Operative time was 437 min (console time: 159 min), and blood loss was 453 mL.

No major postoperative complications were observed, and the patient was discharged on postoperative day 19. The pathological diagnosis was high‐grade invasive urothelial carcinoma (pT2, LVI1, RM0) (Figure 2b).

## 3 Discussion

The prevalence of urothelial carcinoma is higher in solid organ recipients, including KTRs [3].

The cause is thought to be related to immunosuppressant therapy and viral infections [4]. Given this background, it is anticipated that the number of cases in which robot‐assisted surgery is considered for KTRs will increase. Although several reports have suggested the safety and usefulness of RARC for a KTR, no comprehensive research has been reported.

Surgery for patients who have undergone multiple pelvic or abdominal procedures not limited to kidney transplantation is complex. We performed a RARC on a patient who had undergone three prior kidney transplantations.

Anticipating altered pelvic anatomy due to the three previous transplants, we initiated the surgery in an area where normal anatomical structures remained intact. By proceeding with the procedure in regions unaffected by prior surgeries and gradually expanding the surgical field, we were able to reliably identify the ureter of the transplanted kidney. In cases with a history of multiple surgeries, we believe it is crucial for safety to modify standard surgical protocols and proceed with the operation starting from areas least affected by previous interventions.

Conversely, we encountered significant challenges in areas where severe adhesions and tissue induration surrounded the vascular anastomoses of the transplanted kidney. Having anticipated this preoperatively, we elected not to perform a pelvic lymph node dissection. Studies on RARP in KTRs frequently report the omission of lymph node dissection due to concerns regarding the risk of vascular injury [5].

Although we simultaneously removed the third transplanted kidney during this procedure, we performed that specific part of the operation via open surgery. While the anastomotic site between the transplanted kidney vessels and the common iliac vessels could be visualized robotically, we judged the risk to be too high and opted for an open approach for that portion of the procedure.

There are likely varying opinions regarding the optimal surgical approach for such complex cases. With the widespread adoption of robotic surgery, robot‐assisted procedures have become a technique in which many urologists are now proficient. Reports exist of RARC and intracorporeal urinary diversion (ICUD) being performed on KTRs; thus, RARC may be a safe and effective procedure for this patient population [6, 7].

We believe that selecting a technique with which the surgeon is proficient is crucial for ensuring safety when dealing with complex cases. In this instance, robot‐assisted surgery proved highly effective, particularly when initiating the procedure on the left side via the native ureter and subsequently advancing the dissection within a confined surgical field to the level of the seminal vesicles and the dorsal aspect of the prostate. Consequently, we were able to safely perform the cystectomy using the robotic approach.

However, it is essential to obtain thorough informed consent beforehand, ensuring the patient understands that a conversion to open surgery might become necessary depending on the intraoperative situation.

## Ethics Statement

This single‐patient case report is exempt under our institutional policy.

## Consent

Written informed consent for publication, including images, was obtained from the patient.

## Conflicts of Interest

The authors declare no conflicts of interest.

## Data Availability

Data sharing not applicable to this article as no datasets were generated or analyzed during the current study.
